# The influence of proline isomerization on potency and stability of anti-HIV antibody 10E8

**DOI:** 10.1038/s41598-020-71184-7

**Published:** 2020-08-31

**Authors:** Miklos Guttman, Neal N. Padte, Yaoxing Huang, Jian Yu, Gabriel J. Rocklin, Brian D. Weitzner, Michele Scian, David D. Ho, Kelly K. Lee

**Affiliations:** 1 Department of Medicinal Chemistry, University of Washington, Seattle, WA 98195 USA; 2 Aaron Diamond AIDS Research Center, Columbia University Vagelos College of Physicians and Surgeons, 630 W 168th St, New York, NY 10032 USA; 3 Department of Pharmacology, Northwestern University, Chicago, IL 60611 USA; 4 Department of Biochemistry, University of Washington, Seattle, WA 98195 USA; 5 Institute for Protein Design, University of Washington, Seattle, WA USA

**Keywords:** Biochemical assays, NMR spectroscopy, SAXS, Antibody therapy, Recombinant protein therapy

## Abstract

Monoclonal antibody (mAb) 10E8 recognizes a highly conserved epitope on HIV and is capable of neutralizing > 95% of circulating viral isolates making it one of the most promising Abs against HIV. Solution instability and biochemical heterogeneity of 10E8 has hampered its development for clinical use. We identify the source of 10E8 heterogeneity being linked to cis/trans isomerization at two prolines within the YPP motif in the CRD3 loop that exists as two predominant conformers that interconvert on a slow timescale. The Y_trans_P conformation conformer can bind the HIV gp41 epitope, while the Y_cis_P is not binding competent and shows a higher aggregation propensity. The high barrier of isomerization and propensity to adopt non-binding competent proline conformers provides novel insight into the slow binding kinetics, low potency, and poor solubility of 10E8. This study highlights how proline isomerization should be considered a critical quality attribute for biotherapeutics with paratopes containing potential cis proline amide bonds.

## Introduction

Since their introduction in the market in 1986, monoclonal antibodies (mAbs) have grown to become a dominant platform for biotherapeutics. Through the years, extensive biophysical and structural characterization have paved the way for the development of more effective mAbs through engineering for better properties including antigen binding, effector function, pharmacokinetics, and solution stability^1^. For HIV, mAbs directed against the envelope glycoprotein (Env) present a means for achieving both passive immunity and therapeutic treatment^2,3^. Several antibodies have been shown capable of neutralizing a wide variety of viral isolates, termed broadly neutralizing antibodies (bNAbs)^4^. Antibody 10E8 is one of the most promising bNAbs, which targets the membrane proximal external region (MPER), a highly conserved site on Env^5^. While 10E8 can neutralize more than 95% of viral isolates, its poor solubility has presented challenges in its development as a viable therapeutic mAb^6,7^. 10E8 has also been shown to be heterogeneous by size-exclusion chromatography (SEC), a critical assay for development and characterization of potential therapeutic mAbs, for reasons which have yet to be fully elucidated^7,8^. Bispecific antibodies and re-engineered versions of 10E8 have alleviated many of the solubility issues, but still show apparent heterogeneity by SEC^9^.

Proline residues are unique amino acids in that they can adopt a stable cis amide bond, with a high barrier of rotation that can act as a conformational switch and plays a critical role in the folding and function of many proteins^10^. In the context of a free peptide, the trans amide conformation is energetically favorable and the predominant state in solution. Prolines preceded by aromatic residues have a higher propensity to adopt the cis-conformer as it is stabilized by interactions with the aromatic sidechain^11–21^. Here we demonstrate that the cis/trans isomerization state of a pair of prolines within the complementarity determining region 3 of the heavy chain (CDRH3) of 10E8 is responsible for the apparent SEC heterogeneity observed by multiple studies. More importantly, the proline isomerization state of CDR3 also governs the solution stability of 10E8, as well as its antigen binding properties. This study illustrates how proline isomerization should be considered a potential critical quality attribute for both characterization and engineering of mAbs that, based on available structures, is relevant to many potential therapeutic antibodies.

## Results & discussion

**Figure 1:**
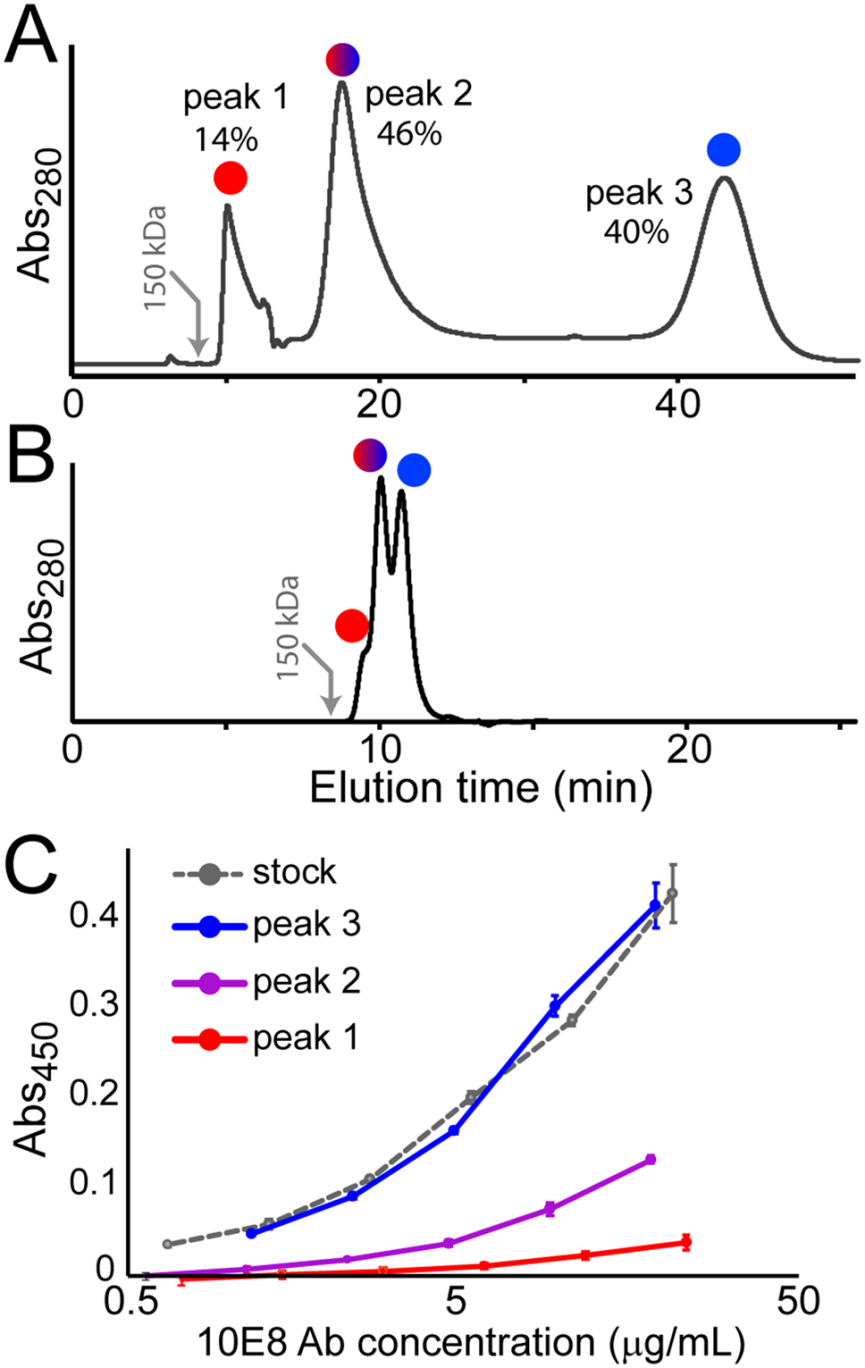
(**A**) SEC trace of Ab 10E8 showing the three isomeric peaks using a Sepax SRT-300 (**A**) or SRT-C 300 column (**B**). The later was designed to minimize secondary hydrophobic interactions with the column matrix. The relative percentage based on UV absorbance is shown above each peak. Gray arrows indicate the expected elution time based on molecular weight standards. (**C**) ELISA of gp41 binding of each isolated SEC peak showing that peak 3 has the highest affinity for gp41, while peak 1 shows weak binding.

To investigate the apparent heterogeneity of 10E8 we performed SEC under a series of conditions. As reported previously^7,8,22^, 10E8 showed elution profiles with multiple peaks. When performing SEC with a monolayer coated silica matrix (Sepax, SRT), three baseline resolved peaks (here denoted as “SEC peaks 1–3”) were observed that eluted much later than expected based on standard protein molecular weight markers (Fig. 1A). Using a lay-down monolayer coated silica SEC column (Sepax, SRT-C) alleviated the extensive column retention, but still showed three partially resolved peaks (Fig. 1B). Previous studies have shown that the SEC resolved 10E8 peaks corresponded to single IgGs that are in a slow dynamic equilibrium^7,8,22^. To confirm that our 10E8 was similarly behaved we performed SEC with multi-angle light scattering (MALS) and observed three peaks, each of which had a molecular mass near 150 kDa consistent with an intact IgG, as reported previously (Fig. S1). To quantitatively investigate the dynamic equilibrium among the isomers, we performed preparative SEC to collect individual species and reinject them over SEC after a range of delays to measure the kinetics of isomerization. When the last eluting peak (“peak 3”) was reinjected, after 30 min it only partially re-equilibrated, but after 2.5 h it yielded a SEC profile identical to the starting material (Fig. S2). Conversely, peak 1 or peak 2 reinjected after 12 h also yielded an identical SEC profile as the starting material. From the time points collected, the rate of inter-conversion at 25 °C from isolated peak 3 back to the equilibrium distribution was found to be 0.00034 ± 0.00004 s^-1^, corresponding to a half-life of 34 min.

To test for any biologically relevant phenotypic differences among the SEC resolved isomers we isolated each fraction and performed a rapid ELISA against HIV gp41 (see “Methods”). Being mindful of the isomerization kinetics, we carried out the protein concentration step and the primary incubation with the antigen at 4˚C for only a short duration. Concentrations of 10E8 isomers were quantified by quantitative western blot after the ELISA with the remaining concentrated protein (Fig. S3, see “Methods”). Interestingly, SEC peak 3 had by far the most binding activity for gp41, followed by moderate binding with SEC peak 2, and nearly no binding with SEC peak 1 (Fig. 1C). Because some degree of isomerization could take place under the experimental conditions, the residual binding of SEC peak 1 may be attributed to low levels of re-equilibration. These data reveal that the SEC resolved isomers of 10E8 have different biological activity due to structural changes that affect the antigen-binding paratope region.

**Figure 2:**
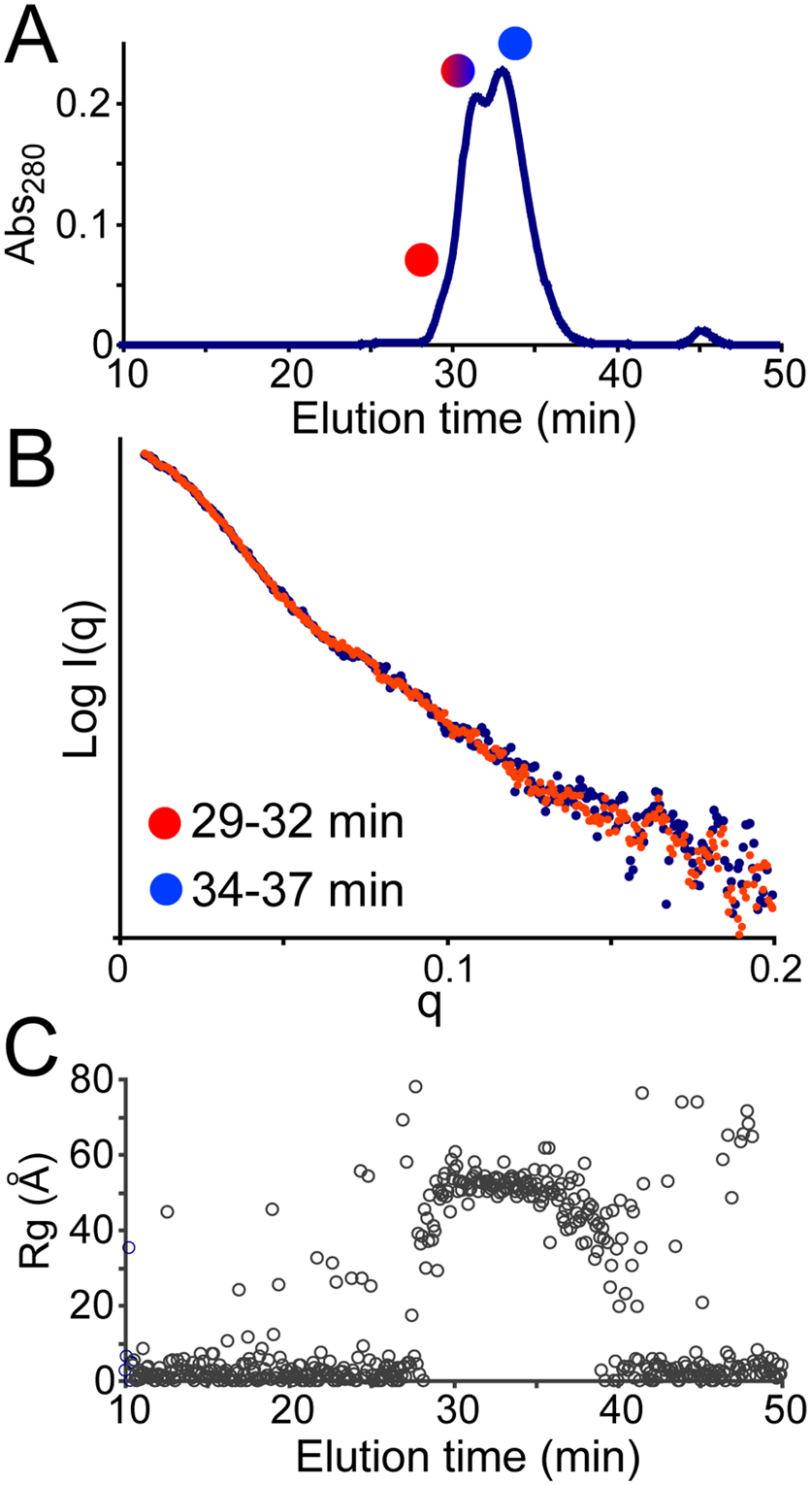
(**A**) 10E8 isomers were partially resolved by SEC over superdex S200 and analyzed online with SAXS. The two halves of the split peak showed no significant differences in their scattering profiles (**B**) and the radius of gyration was consistent for all species (**C**).

Since the oligomeric state of the protein is consistent in all three SEC peaks, and the isomers are in dynamic equilibrium, we suspected that the differences among the isomers are due to conformational differences that interconvert on a slow time scale. To probe for any variations in large-scale conformation we performed SEC with online small angle X-ray scattering (SAXS), which provides a rotationally averaged scattering profile to extract a low-resolution structural information of the 10E8 isomers^23^. Due to limitations with the SEC-SAXS setup, a Superdex S200 (GE Healthcare) SEC column was used, which did not resolve the 10E8 isomers as well as the Sepax SRT columns. However, two major peaks and one early shoulder were still apparent (Fig. 2A). Examination of the two sides of the partially resolved peak showed identical SAXS profiles (Fig. 2B). The radius of gyration (Rg), extracted from the low angle portion of the data also showed no difference across the doublet peak (Fig. 2C). Together this argues against any large-scale conformational differences among the 10E8 isomers.

**Figure 3:**
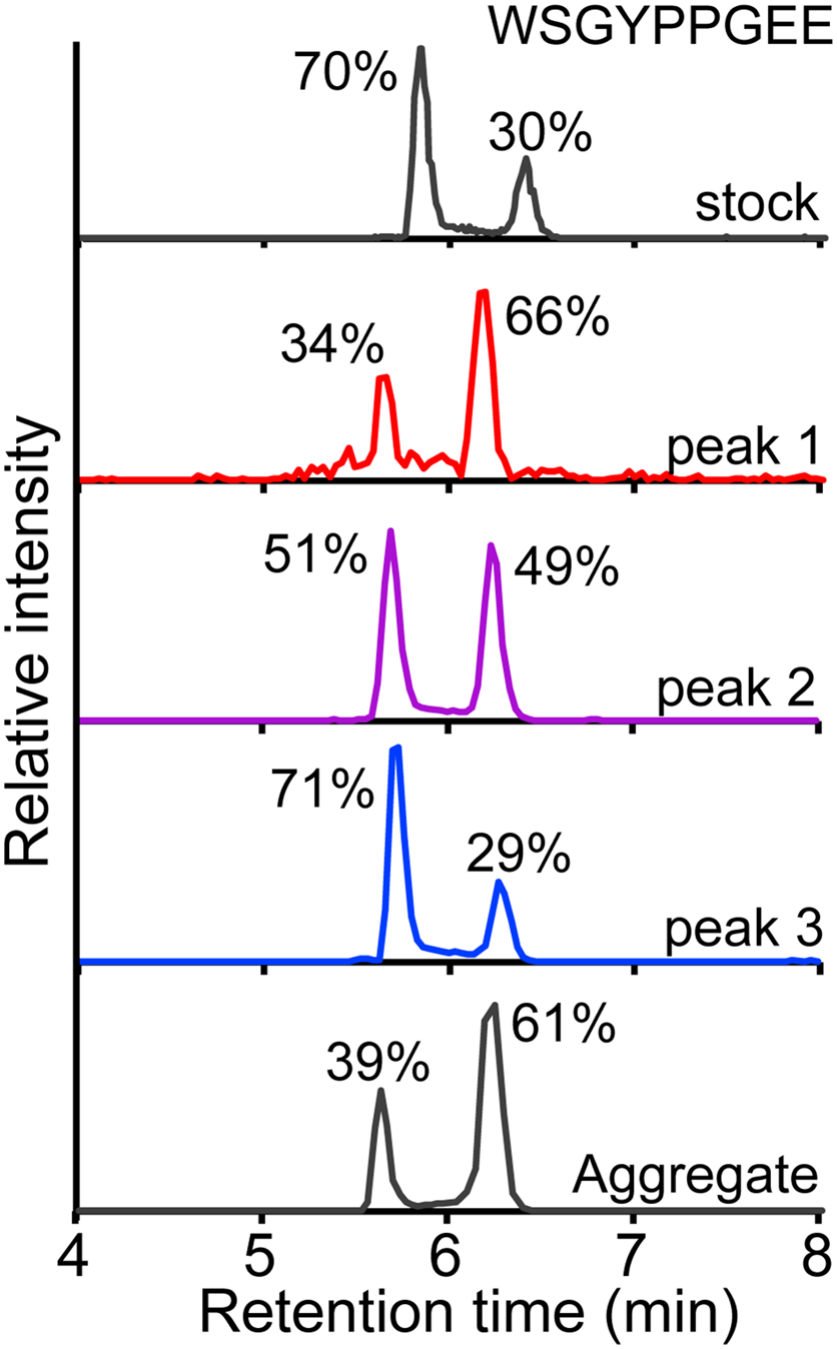
LC–MS of peptic digests of the WSGYPPGEE peptide peak using C18 reverse phase chromatography of either the stock (unfractionated 10E8, top), SEC peaks 1–3, and aggregated 10E8 (bottom). The relative intensities extracted from the LC–MS data are shown above each peak.

Looking back on the isomerization kinetics we noted that the timescale of 10E8 isomerization (3.4e−4 s^−1^) was in the realm generally observed for proline isomerization (3e−2 to 4e−5 s^−1^)^11,15,24–26^, and we suspected this may be ultimately responsible for the distinct SEC peaks. To examine heterogeneity in proline isomerization on a peptide level, we utilized a bottom up LC–MS approach using pepsin digestion. Pepsin is a well-established protease for rapid digestion of proteins to generate a diverse set of peptides that cover a large portion of the target protein^27^. Furthermore, pepsin has a low propensity for cleaving near proline residues thereby increasing the odds of having a peptide with central prolines. Peptides with distinct proline isomerization states can often be resolved by reverse phase chromatography due to differences in their hydrophobicity^24^. Maintaining cold temperatures during pepsin digestion and the LC separation helps to slow cis/trans proline isomerization during sample handling and analysis. Pepsin generated peptides were identified by LC–MS/MS covering 99% of the heavy and 98% of the light chain primary sequence. While nearly all the pepsin generated peptides eluted from the C18 column as a single peak, there were two peptides that stood out showing two distinct retention times. Peptides WSGYPPGEE and WSGYPPGEEY (residues 100b–100j and 100b–100k using Kabat numbering) from the CDRH3 showed two well-resolved peaks (Fig. 3), both of which had the exact mass and MS/MS fragmentation spectra matching the sequence (Fig. S4). Several other proline containing peptides from the remaining 27 prolines in the Fab were identified, but none of them showed multiple retention times (Fig. S5).

The rapid pepsin-LC–MS assay was repeated on the fractionated SEC peaks to assess for differences in proline isomerization within native 10E8. Interestingly, SEC peak 1 showed that the early eluting peak of peptide 100b–100j was half as intense as the late-eluting peak (Fig. 3). Conversely SEC peak 3 showed the opposite trend with 70% of the total signal in the early eluting peak. Meanwhile, SEC peak 2 showed equal intensities for both early and late eluting forms. The remaining peptides throughout the protein sequence outside of the CDRH3 were indistinguishable from the three SEC peaks. Thus far, data implicates the YPP motif in CDRH3 as being the main feature responsible for differences between the SEC resolved 10E8 isomers. We also examined the pepsin-LC–MS profile of 10E8 aggregates, which form under long-term storage and heat-stress^7,8,28^. The aggregated material had a much larger population (61%) of the late eluting C18 peak for peptides at the YPP region (Fig. 3, bottom panel).

To verify that the prolines were directly related to the SEC peak splitting we generated and examined point mutations within the YPP region. The apparent SEC heterogeneity was completely alleviated by removal of either P100f. (Y**P**P) or P100g (YP**P**) (P120 and P121 in AHO numbering) (Fig. S6A). To assess whether the proline mutant constructs were still active we compared their abilities to neutralize HIV infection in the context of the 10E8/iMab construct as described previously^9^. While the WT maintained strong neutralizing activity, the activity of both proline mutants was severely diminished (Fig. S6B).

**Figure 4:**
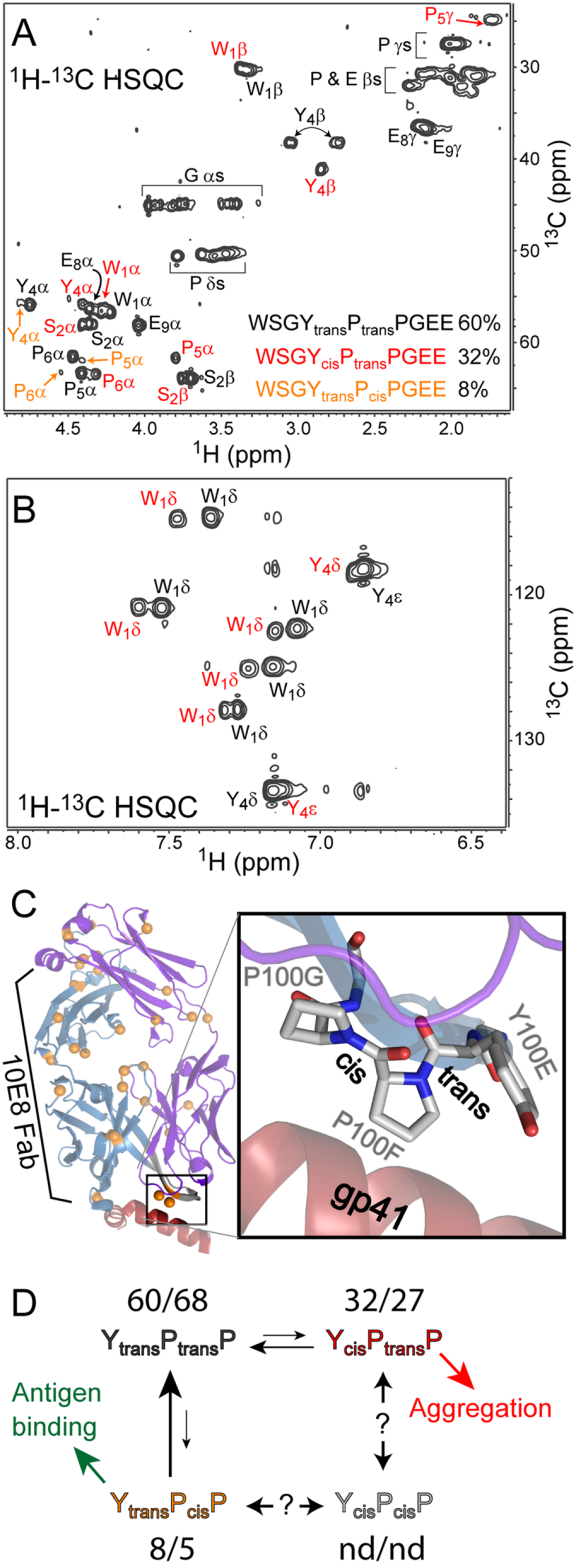
(**A**) 2D ^1^H–^13^C HSQC NMR spectra of 10E8 CDRH3 peptide WSGYPPGEE at pH 7.0 with assigned cross peaks labeled. The peaks corresponding to the peptide conformations with Y_trans_P_trans_P, Y_cis_P_trans_P, and Y_trans_P_cis_P are shown in black, red, and orange, respectively. The aromatic region of the spectrum is shown in (**B**) with the same labeling, though cross peaks for the Y_trans_P_cis_P conformation could not be assigned. The populations estimated from relative peak intensities are shown in the inset. (**C**) Structure of the 10E8 Fab (PDB 4G6F) with all 29 prolines in the Fab shown as orange spheres. The zoomed view of the CDRH3 loop contacting the gp41 shows key residues and the isomeric state of each proline peptide bond. (**D**) Schematic of the various conformers of 10E8 defined by the proline isomerization state within CDRH3. Numbers above each of the states reflect the percentage present by NMR at either pH 7.0 or 5.5. The cis/cis conformation was not detected (nd). The trans/trans conformation is the most abundant in solution, the cis/trans conformation that is susceptible to aggregation, while the trans/cis is the conformation that is binding competent for gp41.

We next sought to identify the specific cis/trans proline isomers within the CDR3 region. The synthetic WSGYPPGEE peptide analyzed by LC–MS also showed the same double peak as was observed in the pepsin digests of 10E8 (Fig. S7). When reinjected, both the early and late eluting peptide peaks converted back to the same two peaks and the re-equilibration kinetics were similar to the kinetics measured for the intact 10E8. Therefore, it appears this amino acid sequence has a high propensity of isomeric heterogeneity regardless of the surrounding higher order structure. The peptide was then examined by nuclear magnetic resonance (NMR), to identify the specific cis/trans proline isomeric states within the coexisting peptide conformers. The ^1^H–^13^C HSQC spectra revealed more than double the number of expected resonances for a single conformer. The aromatic region also showed approximately twice the expected number of cross peaks, indicating that at least two predominant conformations were present. From a series of NMR experiments (see “Methods”), many of the backbone and sidechain resonances could be assigned. The most abundant conformer (60% abundance by peak intensity) showed strong NOEs for Y_100e_ Hα-P_100f_Hδ and for P_100f._ Hα-P_100g_ Hδ, consistent with trans peptide bonds^11,29^. This was also confirmed by the proline chemical shifts, particularly for the Cγ (27.44 ppm, with 27.45 ± 0.86 ppm expected for trans prolines^30^). The second major population (32% abundance, Fig. 4A,B in red), still had a strong Hα-Hδ NOE at P_100f._-P_100g_, but had a strong Y_100e_Hα-P_100f_Hα NOE, consistent with a Y_cis_P_trans_P conformation. This was also confirmed by the chemical shift of this Y_100e_Cγ of 24.89 (24.41 + /- 0.74 expected for cis prolines)^30^. Lastly, a minor population (~ 8% abundance, Fig. 4A,B in orange) was observed which had a strong Y_100e_Hα-P_100f_Hδ NOE, but only showed a P_100f_Hα-P_100g_Hα NOE, consistent with a Y_trans_P_cis_P conformation. To assess whether pH had a significant effect on proline isomerization state in solution, a second series of NMR experiments were conducted and assigned at pH 5.5. Overall, the pH 5.5 data set showed very similar profiles and ratios of the different proline isomers (Fig. S8).

From the available data we conclude that it is the Y–P bond isomerization, not the P–P bond that governs the retention time observed by LC for the WSGYPPGEE peptide and the elution time by SEC for the intact 10E8. The predominant conformer in solution is the Y_trans_P_trans_P isomer at both pH 7.0 and 5.5, which corresponds to the predominant earlier eluting peptide observed by pepsin-LC–MS (Fig. 3). It is interesting that only two peaks were observed for the WSGYPPGEE peptide by LC–MS despite that there should be a third population (Y_trans_P_cis_P) accounting for 5–8% of the signal, as seen by NMR. This is either due to either 1) the Y_trans_P_trans_P and Y_trans_P_cis_P isomers having very similar hydrophobicities and not getting resolved by C18 chromatography; or 2) the P–P bond isomerizing much faster than the Y–P bond and thus we only observe the average retention time by Pepsin-LC–MS. Given that the Y_trans_P_cis_P isomer showed distinct cross peaks by NMR, the interconversion for the P–P bond is still relatively slow, otherwise the Y_trans_P_cis_P and Y_trans_P_trans_P isomers would have been observed as a single averaged state. Furthermore, the backbone Hα resonances were similar for Y_trans_P_trans_P and Y_trans_P_cis_P isomers, but very different for the Y_cis_P_trans_P (Fig. 4A) indicating a distinct structure and thereby likely a different hydrophobicity. Therefore, the first eluting peak observed by LC–MS for the WSGYPPGEE peptide should correspond to both the Y_trans_P_trans_P and Y_trans_P_cis_P isomers, while the later eluting peak is solely the Y_cis_P_trans_P isomer. The alternative hypothesis, that the Y_cis_P_trans_P and Y_trans_P_cis_P isomers have identical elution times is much less likely as they differ at both amide bond positions.

The cis/trans isomeric state governing secondary interactions with SEC matrices also explains the multiple peaks observed by SEC for intact 10E8. In the context of a full IgG, either Fab arm can have different proline isomerization states within the YPP region, specifically the Y–P bond, and this explains the three peaks observed by SEC. SEC peak 1 corresponds to having both Fabs in the Y_cis_P state, peak 3 with both Fabs as Y_trans_P, and peak 2 with one of each. Upon rapid pepsin digestion the 10E8 IgG, SEC peak 3 should yield only peptides that have the Y_trans_P isomer. Due to re-equilibration occurring during fraction collection and pepsin-LC–MS analysis (which requires approximately 15 min), there is a significant (~ 29%) portion that has converted to Y_cis_P. We note that this type of interconversion is also observed when reinjecting the early eluting Y_trans_P synthetic peptide; after approximately 5 min it has converted to 18% Y_cis_P (Fig. S7). Interestingly, the available crystallographic data shows the CDRH3 region in a Y_trans_P_cis_P state when bound to HIV gp41. Since only SEC peak 3 had a high affinity for gp41, we infer that the SEC peak 3 likely contains both the Y_trans_P_trans_P and Y_trans_P_cis_P isomers. We note that both LC–MS of the peptides and SEC of the intact IgG is not able to resolve the isomerization of the P–P bond. Since the Y_trans_P_trans_P and Y_trans_P_cis_P isomers are apparently not resolved we currently cannot determine how much of the intact 10E8 Fab is in the binding competent Y_trans_P_cis_P isomeric state. However, at the least we can conclude that all of SEC peak 1 (14% of the total) and half of SEC peak 2 (23% of the total) are in the Y_cis_P isomeric state, accounting for a total of 37% of the Fab portions of 10E8.

Together these data indicate that the Y_100f_–P_100g_ bond isomerization, rather than P_100g_–P_100h_, govern secondary SEC interactions that have been observed in numerous studies^7,9^. Recently it was reported that the SEC peak splitting was alleviated by running in PBS at pH 10.55 and supplementing with 100 mM Arg^8^. The effect of high pH is interesting as the average sidechain pKa of tyrosines in proteins is 10.3 ± 1.2 ^31^, and thus it may abolish the secondary hydrophobic interaction between the YPP region and the column matrix either directly or by perturbing the kinetics of cis/trans isomerization at the Y–P bond which then alter the SEC secondary interactions.

### Biological implications of cis/trans proline isomerization

The structural heterogeneity within CDRH3 of 10E8 that we describe here provides a new rationale for many previous observations. 10E8 has significant breadth (> 95%), but does not achieve the levels of potency (IC_50_) that has been observed for other bNAbs^32^. This limited potency has been attributed to steric occlusion near the gp41 binding site due to its membrane proximity^22^. In light of the current data, we propose that it is the barrier of proline isomerization at CDRH3 that is severely limiting 10E8 antigen binding. Based on the NMR data, in solution the 10E8 CDRH3 primarily adopt a Y_trans_P_trans_P and Y_cis_P_trans_P conformation. Only ~ 8% of the 10E8 Fab is in the binding competent Y_trans_P_cis_P configuration observed in the crystal structure (Fig. 4C)^6^. Therefore, > 90% of the population must undergo conformational sampling until the prolines re-equilibrate to adopt the correct conformation to bind (Fig. 4D). Though the isomerization kinetics were measured at 25 °C, the slow rates of isomerization are still expected to have significant effects in vivo. Based on the Arrhenius equation and an estimated activation energy of 20 kcal/mol^11^ the isomerization rate is expected to be 1.7e−3 s^−1^ (6.8 min t_1/2_) at 37 °C, which can still severely limit the amount of active (binding competent) 10E8 in vivo. Since both the SEC and C18 LC peak splitting are governed by the Y_100f_–P_100g_ bond, from the current data we cannot estimate the isomerization kinetics of the P_100g_–P_100h_ bond within intact 10E8. Previous studies have shown that the barrier of Pro-Pro isomerization is actually higher than Tyr-Pro, and therefore the Pro-Pro isomerization kinetics are expected to be even slower than the ones measured here^15^. However, proline isomerization kinetics can be highly context dependent and requires further investigation^11,15,16^.

The major therapeutic potential of 10E8 has spurred antibody engineering efforts to improve solution stability and potency^7,28,33^. Addition of a disulfide bond that included mutation of Y_100e_ (the Y in the YPP motif in CDRH3) to cysteine alleviated the 10E8 SEC peak splitting, however at the cost of decreased breadth and potency^7^. In the case of Y_100e_C mutation, the resulting Cys-Pro motif will likely impact the propensity to form the cis amide conformation and the mutation may thus alter the kinetics of the proximal P100f.-P100g bond isomerization and alter antigen binding. Other mutations within CDRH3, including mutations at Ser_100c_ directly N-terminal to the YPP motif have been shown to dramatically enhance 10E8 potency^28^. We argue that these modifications are influencing the cis/trans isomerization states of the YPP, likely favoring the Y_trans_P_cis_P isomer to enhance antigen binding, and thereby improving potency. Previous studies have also implicated mutations within CDRH3 relating to membrane interactions with 10E8^5,22^, which is the same region shown to be responsible for membrane interactions with similar MPER targeting antibodies^34–37^. In light of our new data, it is possible that these mutations in 10E8 offset proline isomerization kinetics and underlie the observed changes in membrane binding. Alternatively, it is possible that membrane interactions with 10E8 favor formation of the Y_trans_P_cis_P conformation, and thereby promote formation of the binding competent isomer. This would explain why 10E8 compared to other MPER antibodies (2F5 and 4E10) has weaker affinity in vitro, but is far more potent in neutralization assays^5,6^.

An interesting observation of this study was that both SEC peak 1 and the aggregated 10E8 material contained a high content of the Y_cis_P isomer. Furthermore, SEC peak 1 showed no binding to gp41. This suggests that the Y_cis_P_trans_P conformation that accounts for approximately 30% of what is present in solution is not only binding incompetent, but also aggregation prone (Fig. 4D). The later elution from C18 is consistent with the Y_cis_P isomer being more hydrophobic, which could be responsible for driving self-association. Future efforts to develop 10E8 for therapeutic use should consider the influence on the proline isomerization state within CDRH3, while maintaining critical contact residues with the antigen. Alteration of nearby, non-contacting, residues to disfavor a cis amide conformation at Y_100e_–P_100f._ bond, while favoring the cis conformation at the P_100f._–P_100g_ bond are expected to decrease aggregation propensity while increasing potency. Alternatively, mutations that accelerate the bond isomerization at the P_100f._–P_100g_ may also benefit 10E8 potency.

Although the peak splitting that motivated the current study was a caveat that hampered aggregation studies, in this case the non-specific interaction with the SEC material was serendipitous for revealing a critical quality attribute for a potential biotherapeutic. There may be several systems for which SEC with secondary hydrophobic interactions may be useful for assessing conformational heterogeneity even beyond proline isomerization. More tailored analytical approaches should also focus on tracking proline isomerization, especially when they are present in the CDR regions. The pepsin-LC–MS assay described here is nearly identical to the approach used for Hydrogen/deuterium exchange studies^38^, and presents a versatile tool for tracking proline isomerization that could be easily adapted to just about any protein of interest. Recently, the feasibility of examining intact IgGs by NMR has been demonstrated^39^, and with the appropriate isotopic labeling this could present a very powerful method to directly probe proline isomerization within intact IgGs. However, the material and equipment requirements for carrying out this analysis was well beyond the scope of this study.

During the submission stage of this manuscript Masiero et al., reported their findings of proline isomerization within a trispecific mAb that included 10E8v4 Fab (the same 10E8/iMab construct examined here) and the effect was localized to the same YPP motif we have implicated here^40^. Based on molecular dynamics simulations the authors concluded that the two observed conformers reflect the Y_trans_P_trans_P and Y_trans_P_cis_P forms. In contrast, our NMR characterization of the 10E8 peptide together with the rapid pepsin-LC/MS assay directly shows that the Y_cis_P_trans_P and Y_trans_P_trans_P are actually the predominant conformers in solution, and therefore it is primarily P100f., and not P100g, that governs the anomalous size-exclusion elution times. Masiero et al. report an association rate of 4,500 and 46,800 s^-1^ M^−1^ for the slower and faster-binding conformer. The faster of these two association rates is still relative slow compared to typical antibody-antigen interactions, indicating an additional conformation rearrangement prior to binding^41^. We hypothesize that the association rate could be limited by need for isomerization of Y_trans_P_trans_P to Y_trans_P_cis_P and any way of favoring the cis conformer at P100g could potentially further improve the potency of 10E8. The tenfold difference in association rates is consistent with our rapid ELISA binding studies, where additional care was taken to minimize the effects of re-equilibration. In light of the evidence that it is generally the association rate of antibody-antigen interactions that correlates strongly with neutralization potency^42,43^, and in contrast with the conclusions of Masiero et al., we argue that the presence of the Y_cis_P_trans_P conformer is significantly detrimental to potency and any efforts to re-engineer the CDR3 to favor the ideal conformer, potentially through fine tuning of aromatic-proline interactions^21^, are warranted.

### How common of a problem is this for biotherapeutics?

To assess whether cis proline isomers are relevant to antibodies-antigen interactions in general, we surveyed the PDB for cis prolines within the variable regions (see “Methods”). Previous surveys suggested that cis amides at prolines can occur in nearly any sequence context, but are most common at prolines preceded by aromatic residues^16,21,44^, but it is unclear how commonly this occurs within the paratopes on immunoglobulins. From a survey of all the Fab domains within the PDB we find that 63% of the entries contained at least one cis proline in either the light or heavy chain (1,637 of 2,582 entries) CDRs (Table S1). 92% of these occurred specifically at P136 (AHO numbering; equivalent to P95 with Kabat numbering) in the kappa light chain, and 7% were throughout the CDRH3. When considering the isomeric state of all prolines within the CDRs 15% were cis. However, excluding the cis proline at P163, common in kappa light chains, the prevalence of cis proline in the CDRs is only 2%, occurring most frequently within CDRH3 and CDRL3. Though seemingly rare, cis prolines were still present within the CDRs of more than half of the known Fab structures, arguing that proline isomerization is a highly relevant factor to consider for engineering and characterizing antibodies.

The importance of cis/trans proline isomerization has been appreciated for decades in protein folding and function^10^, but has only recently become appreciated for antibody development. Binding studies of an effective monoclonal antibody against the human papilloma virus revealed that it recognizes a cis proline conformer that is only present at 10% in the solution structure of the viral E7 domain^45^. The isomerization at this proline occurs on a minute timescale, which explains the slow association rate and highlights that vaccine design efforts will need to utilize immunogens with the ‘correct’ proline isomer to promote effective antibody development. More recently, distinct proline isomers within CDRH3 loop of antibody 9E5 were found between the unbound and antigen-bound structures. Further binding studies were consistent with the cis isomer being the faster-binding competent form, but only present at a lower population^46^. Together with the importance of proline isomerization we, and others, pinpoint in mAb 10E8, these studies suggest a strong need to consider proline isomerization as a critical factor for the design and characterization of therapeutic antibodies and protein therapeutics in general.

## Methods

### SEC and MALS

SEC was performed on Sepax SRT-SEC or SRTC SEC (5 µm, 300 Å, 4.6 × 300 mm with a 4.6 × 50 mm matching guard column) at a flow rate of 0.35 mL/min in 150 mM sodium phosphate pH 7.0, 0.02% sodium azide using an Agilent 1,260 HPLC system. Injections were 50 µL of a 1 mg/mL solution in PBS pH 7.0. For kinetic measurements, fractions were collected manually during peak elution and reinjected at a specific timepoint using identical run methods. MALS measurements were performed using the same SEC setup, but with a light scattering detector (miniDawn Treos, Wyatt Instruments) and a refractive index detector (TRex, Wyatt Instruments) online. Chromatograms were aligned, integrated and the MW calculated using ASTRA (Wyatt Instruments), assuming a refractive index of 0.183 reflecting a weighted combined contribution of protein 0.185 and glycan 0.146^47^.

### SAXS

SAXS with online SEC was collected at beamline 4–2 at SSRL as described previously^48^. 100 µL of a 1 mg/mL 10E8 solution was resolved over a GE Healthcare Superose 200 Plus column at 50 µL/min in phosphate buffered saline (PBS) pH 7.4 with 0.02% sodium azide. The flow from the column was passed through a UV detector followed by a capillary 0.1 mm where 1 s X-ray exposures were collected every 4 s. The merged scattering patterns were processed with sastools^49^ and analyzed using the ATSAS software suite^50^. The reported radius of gyration was estimated using the Guinier method analyzed using Primus^51^.

### LC–MS

Fractions collected from SEC were immediately mixed with an equal volume of 0.2% formic acid, 0.1% TFA and 200 mM tris 2-carboxyethyl phosphine (TCEP) for a final pH of 2.5 and flash frozen in liquid nitrogen. Samples were thawed and injected over a Waters HDX Manager with an inline custom packed pepsin column. Peptides were trapped and resolved over BEH C18 column (1 × 100 mm 1.7 µm 130 Å) using linear gradient of 5 to 35% B (A: 0.1% FA, 0.025% TFA, 5% ACN; B: ACN with 0.1% FA) over 10 min and analyzed on a Waters Synapt G2-Si mass spectrometer. The injection loop, pepsin column, and C18 columns were kept at 1 °C as outlined for previous HDX-MS studies^52^. MS/MS data were collected using a combination of MS^E^ and data-dependent acquisition (DDA). Peptides were identified using protein prospector^53^ and ProteoLynx Global Server 3.0 (Waters) using score thresholds of 15 and 7, respectively. The aggregated 10E8 sample was recovered from a stock that had been sitting at 4 °C for months by centrifugation at 14 k rpm for 15 min, forming a visible white pellet. The pellet was resuspended in 30 µL of cold 6 M GndHCl, diluted to 350uL in SEC buffer, and immediately analyzed by LC–MS with online pepsin digestion just as done for the other SEC samples. To measure re-equilibration kinetics with peptides, 60 µL of the apex of each peak was manually collected from the LC gradient, frozen in liquid nitrogen, thawed, diluted threefold in 0.1% formic acid, and analyzed by the same LC–MS method, but without the pepsin column inline.

### Rapid ELISA

ELISA plates were first prepared by immobilizing 0.2 µg of recombinant gp41 (AIDS reagent #12027), and blocked with 2% BSA in 20 mM Tris, 150 mM NaCl pH 8.0 (TBS). Fractions from the SEC were collected on ice and the rapidly concentrated using microcon 30 kDa spin filters (Amicon) at 4 °C, which took approximately 15 min. The proteins were then immediately added to the wells of the ELISA plate and incubated for 30 min at 4 °C. Wells were washed three times in TBS with 0.02% tween 20 (TBSt), probed with secondary (anti human IgG-HRP, 1:5,000 dilution, Pierce). The remaining volume of concentrated 10E8 fractions was quantified by western blot probed with anti-human HRP secondary antibody (1:12,000 dilution) (Fig. S3). Quantitation of proteins was performed in image J relative to the clean starting 10E8 sample that was quantified by Abs_280_.

### Nuclear magnetic resonance

A 4 mM solution of peptide WSGYPPGEE was prepared in 50 mM sodium phosphate pH 7.0 or 5.5, 10% D_2_O, 0.02% sodium azide containing 0.1 mM 4,4-dimethyl-4-silapentane-1-sulfonic acid (DSS). All spectra were collected at 25 °C on a 499.73 MHz Agilent DD2 spectrometer equipped with a 5 mm triple-resonance ^1^H(^13^C/^15^N), z-axis pulsed-field gradient probe head. Initial ^1^H spectra were collected in 10% D_2_O, after which the sample was exchanged into deuterated buffer by two rounds of drying by centrifugal evaporation and resuspension in 99.9% D_2_O (Cambridge Isotope Labs). ^1^H–^13^C cross peaks in the HSQC spectra were assigned using a ROESY (300 ms mixing time), TOCSY (50 ms mixing time), COSY, and HMBC experiments. Spectra were processed and analyzed with Mnova (Mestrelabs) and the reference for the DSS peak was set to 0 ppm. The HMBC, COSY and TOCSY were used to assign inter-residue correlations, while the ROESY could assign backbone and some side chain connectivity. Overlap at the proline HB and HG regions limited assignments of all sidechain cross peaks, but were sufficient for assigning HA and HD resonances. The relative intensity of the HA_i-1_ –HD_i_ or HA_i_–HA_i-1_ were used to assign cis vs. trans prolines^10,11,29^, which are also consistent with the HA, and carbon chemical shift predicted for cis/trans prolines^30^. All assigned chemical shifts were within one standard deviation from the average values reported in the Biological Magnetic Resonance Data Bank (www.bmrb.wisc.org)^54^.

### PDB survey for cis prolines within CDRs

A survey of 2,582 unique entries in the PyIgClassify database ^55^ was conducted to examine the amide bond (ω) angle for residues 20–47 (CDR 1), 51–82 (CDR2), 102–144 (CDR3) on both chains (AHO numbering, ^56^). Proline residues were considered in the cis form when -90 < ω < 90.

### Mutagenesis and neutralization assays

mAb 10E8 and 10E8-iMab constructs were expressed and purified as previously reported^9^. Mutants were prepared using the Quik-Change II Site-Directed Mutagenesis kit (Agilent Technologies). Size exclusion chromatography (SEC) was used to assess physicochemical homogeneity and to resolve monomers from non-monomeric species. Antibodies (20 µg) were analyzed using an AKTA purifier FPLC (GE Healthcare) as described previously^57^ using either a S200 10/300 GL column (GE Healthcare). Pseudoviruses were prepared as previously described^58^. Virus neutralization was assessed with a single cycle assay using TZM-bl cells and HIV-1 pseudoviruses as described previously^59^.

## Supplementary information

Supplementary Figures.
