# Supplementary material for: The influence of proline isomerization on potency and stability of anti-HIV antibody 10E8
Source: Sci Rep. 2020 Aug 31;10:14313. doi: 10.1038/s41598-020-71184-7 (PMC7458915; doi:10.1038/s41598-020-71184-7)
Supplement: Supplementary file 1 — Supplementary Figures. [file 41598_2020_71184_MOESM1_ESM.pdf]

Supporting Information

The influence of proline isomerization on potency and stability of anti-HIV antibody 10E8

Miklos Guttman[1]*, Neal N. Padte[2], Yaoxing Huang[2], Jian Yu[2], Gabriel J. Rocklin[3], Brian D. Weitzner[4,5], Michele Scian[1], David D. Ho[2], Kelly K. Lee[1]

[1] Department of Medicinal Chemistry; University of Washington, Seattle, WA 98195

[2] Aaron Diamond AIDS Research Center, Columbia University Vagelos College of Physicians and Surgeons, New York, NY 10032, USA

[3] Northwestern University, Dept of Pharmacology, Chicago, IL, 60611

[4] Department of Biochemistry; University of Washington, Seattle, WA 98195

[5] Institute for Protein Design, University of Washington, Seattle, Washington

*Correspondence: mguttman@uw.edu

List of materials:

Figure S1: SEC-MALS analysis of 10E8

Figure S2: SEC re-equilibration kinetics

Figure S3: Western blot quantitation of SEC fractions

Figure S4: MS/MS spectra and drift times of 10E8 peptides

Figure S5: LC-MS traces of all proline containing 10E8 peptic peptides

Figure S6: SEC traces and neutralization assays of proline mutants.

Figure S7: LC-MS traces of synthetic peptide WSGYPPGEE

Figure S8: NMR ^{1}H-^{13}C HSQC spectra at pH 5.5

Table S1: Survey of cis prolines in CDRs in the PDB.

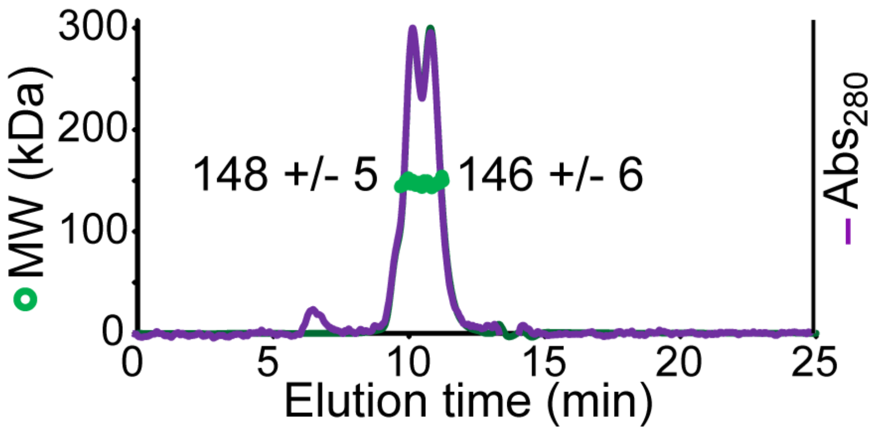

Figure S1. SEC-MALS analysis of 10E8. The UV trace (purple) is shown along with the calculated MW from static light scattering and refractive index (green). The theoretical molecular mass of the 10E8 IgG is approximately 150 kDa.

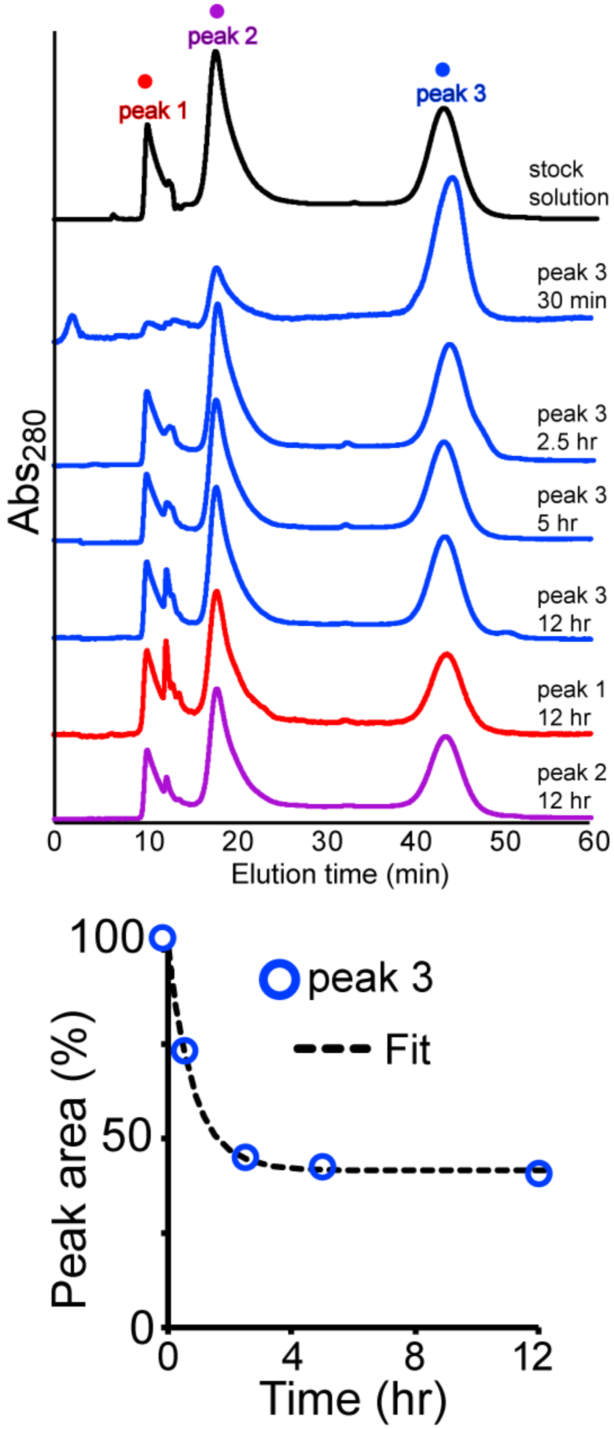

Figure S2. SEC traces tracking the re-equilibration kinetics of the 10E8 isomers. The starting (stock) 10E8 sample is shown at the top. Each trace below is labeled with the fraction that was isolated and re-injected along with the time between collection and reinjection. The kinetics of the re-equilibration of peak 3 is plotted below with dashed lines showing the exponential fit.

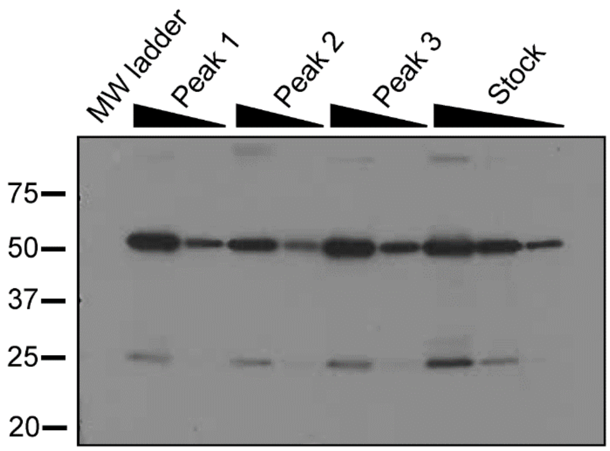

Figure S3. Western blot quantification of SEC peaks. Peaks were collected on ice, rapidly concentrated by spin filtration, and used for ELISA. Small portions of the concentrated peaks were analyzed by western blot as either the concentrated solution (left) or a 1:5 dilution (right). The stock solution was loaded at three dilutions (stock, 1:5, and 1:25) as a reference. Blots were directly probed with a 1:12,000 dilution of HRP-conjugated goat anti-human IgG antibody (Jackson ImmunoResearch Labs, cat# 109-035-088) and developed by chemiluminescence (Western Lightning, Perkin Elmer). Band intensities of both the heavy (50 kDa) and light chain (25 kDa) bands were integrated for all dilutions using image J. The 5-fold dilutions for the HC, and undiluted for the LC (which were in the linear range based on the stock dilutions) were used to calculate the concentrations relative to the stock dilutions, which were quantified by UV_{280}. The quantitative agreement between the heavy and light chain bands for each fraction was within 20% and the average was used to calculate the concentrations present in the ELISA experiments.

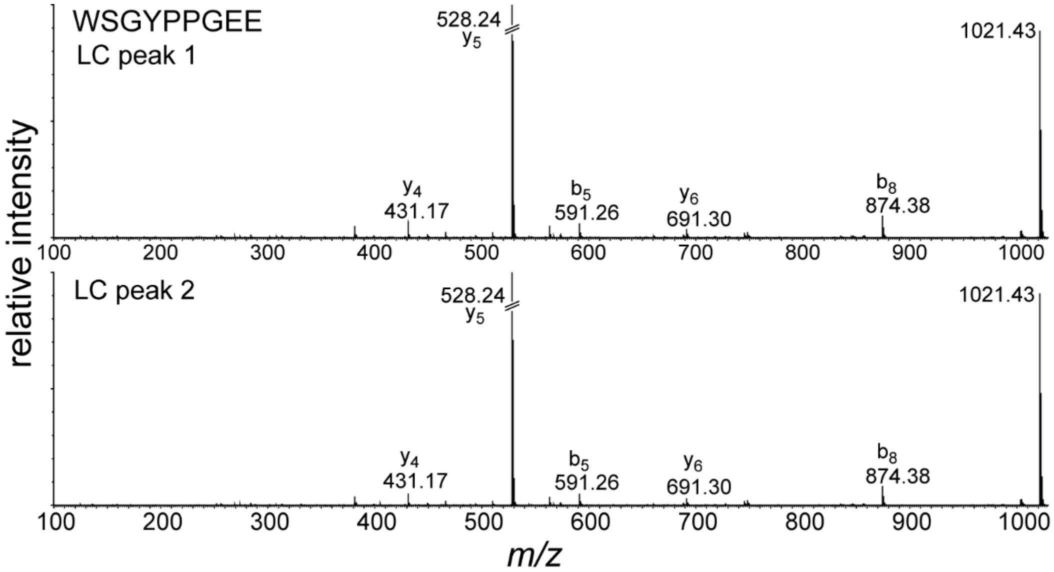

Figure S4. Indistinguishable MS/MS spectra of the first (top) and second (bottom) peaks observed by C18 LC-MS corresponding to the mass of peptide 100b-100j. The major fragment ions are labeled.

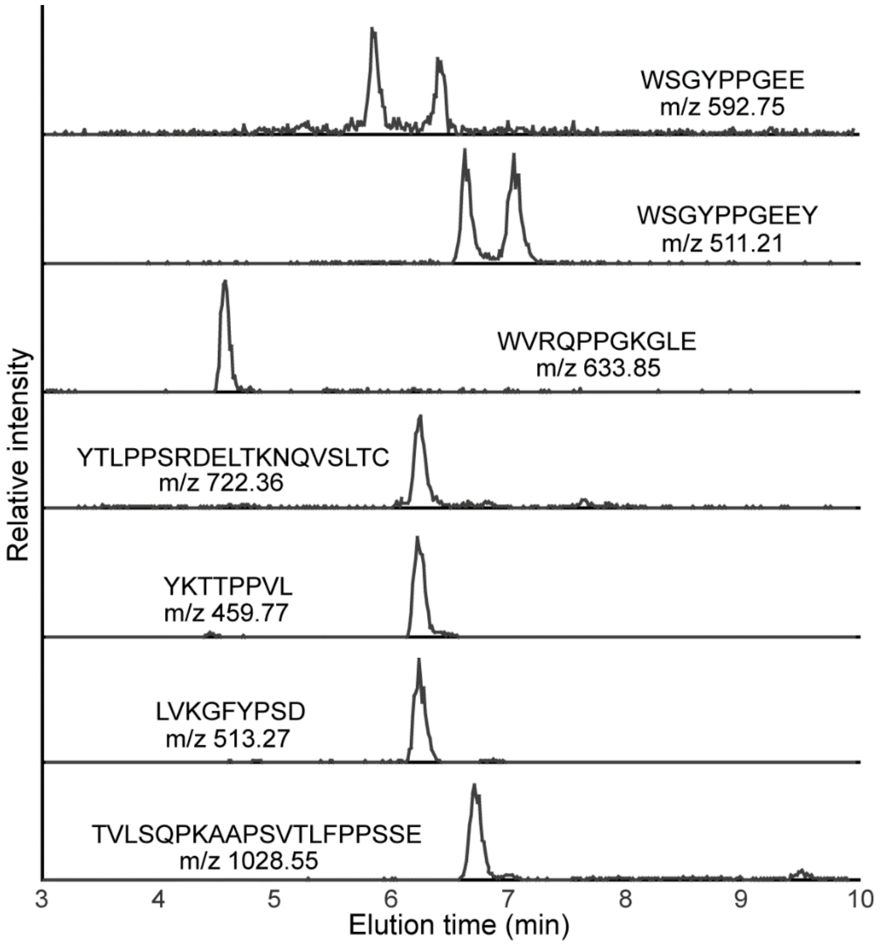

Figure S5. LC-MS extracted ion chromatograms for all proline containing peptides of mAb 10E8. Only the two peptides spanning the YPP region within the CDRH3 showed two resolved LC peaks.

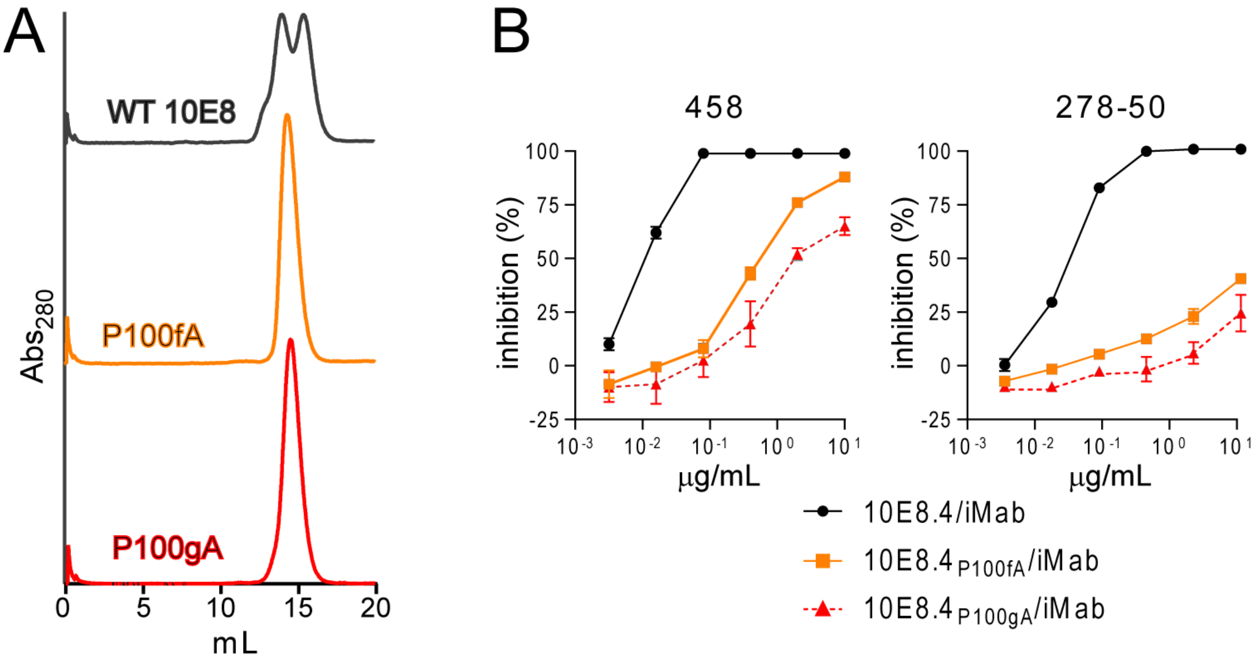

Figure S6: A) Effects of proline mutations within the CDR3 on the SEC traces of 10E8. Mutation of either P100f (orange trace) or P100g (red trace) to alanine abrogates the observed peak splitting. B) Neutralization assays of wild type 10E8/iMab (black) compared to the P100fA (orange) and P100gA (red) mutants.

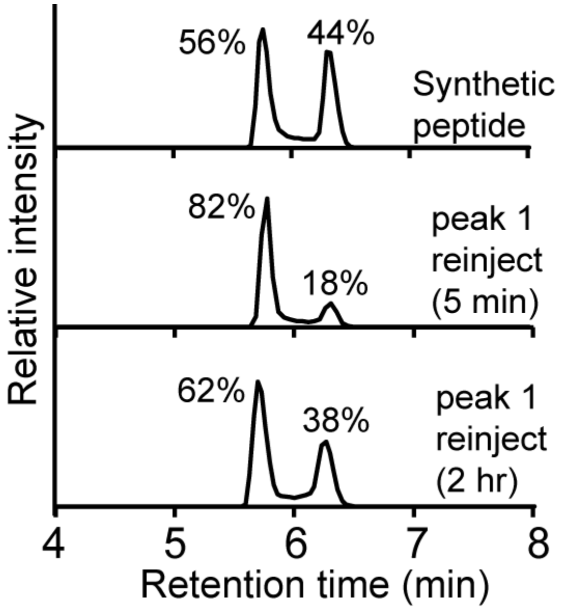

Figure S7: LC-MS extracted ion chromatogram of the synthetic peptide WSGYPPGGEE resolved by C18 reverse phase chromatography. The first eluting peak was isolated, flash frozen, and after a specified amount of time (indicated next to each trace) was thawed, diluted into A buffer and reinjected.

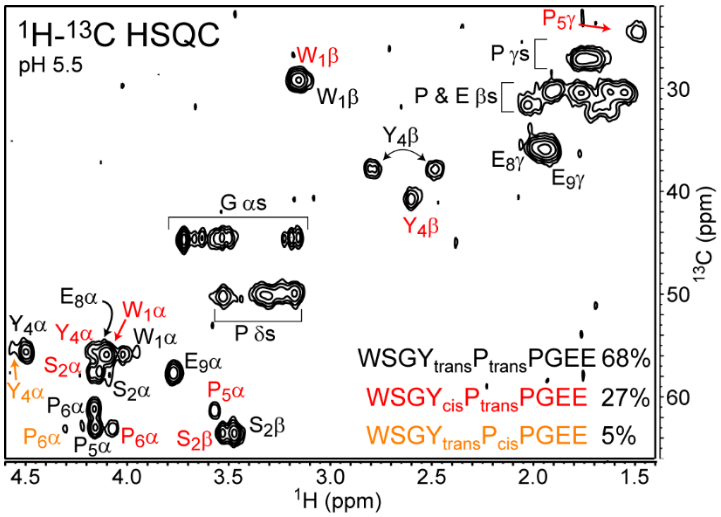

Figure S8: ^{1}H-^{13}C HSQC spectrum of 10E8 peptide WSGYPPGEE in 50mM sodium phosphate pH 5.5. The peaks corresponding to the peptide conformations with $Y_{trans}P_{trans}P$, $Y_{cis}P_{trans}P$, and $Y_{trans}P_{cis}P$ are shown in black, red, and orange, respectively. Relative populations based on cross peak signal intensities for the three observed isomers are shown in the inset.

CDR	number of unique entries with cis proline	Percent of proline in cis
H1	10	3.3
H2	4	0.15
H3	120	13
L1	1	1.3
L2	3	0.03
L3	1499 (66)[a]	76 (12)[a]

Table S1: Frequency of cis prolines within each CDRs in all immunoglobulin structures in the PDB. The percent cis prolines were calculated as the percentage of cis prolines in the CDRs relative to all prolines in the CDRs.

[a] Numbers in parentheses exclude P95, which is a conserved cis proline in kappa light chains.
